# Differential Nicotinic Modulation of Glutamatergic and GABAergic VTA Microcircuits

**DOI:** 10.1523/ENEURO.0298-19.2019

**Published:** 2019-12-03

**Authors:** Yijin Yan, Nicole A. Beckley, Veronica J. Kim, Ryan M. Drenan

**Affiliations:** 1 Department of Physiology and Pharmacology, Wake Forest University School of Medicine, Winston-Salem, North Carolina 27101; 2 Department of Pharmacology, Northwestern University Feinberg School of Medicine, Chicago, Illinois 60611

**Keywords:** nicotinic acetylcholine receptor, dependence, VTA, glutamate, GABA, tobacco

## Abstract

Ventral tegmental area (VTA) neurons receive glutamatergic and/or GABAergic input from other local neurons within the VTA. Nicotinic acetylcholine receptor (nAChR) activity is capable of modulating such intra-VTA transmission, but the mechanisms are unclear. Here, we isolated monosynaptic glutamate or GABA transmission from mouse medial VTA (mVTA) to lateral VTA (latVTA) using pharmacology and optogenetics, and we studied the ability of nicotine to modulate these modes of transmission. The action of nicotine on mVTA to latVTA glutamate transmission was bidirectional; nicotine enhanced glutamate release in half of the recorded latVTA cells and inhibited release in the other half. Nicotine-mediated reduction in glutamate release was reversed by blockade of GABA_A_ receptors. This, coupled with expression data demonstrating coexpression of vesicular glutamate transporter 2 (VGluT2) and glutamate decarboxylase 2 (Gad2) in mVTA neurons, suggests that nicotine is able to stimulate GABA corelease from mVTA VGluT2^+^ neurons. Nicotine had an altogether different effect on mVTA to latVTA GABA release from Gad2^+^ cells; nicotine suppressed GABA release from mVTA Gad2^+^ terminals in nearly all cells tested. Together, these data uncover a complex system of local circuitry in the VTA that is modulated by nAChR activity. These actions of nicotine, which occurred at concentrations of nicotine found in the artificial CSF of cigarette smokers, may play a role in the adaptive response of the reward system to repeated nicotine exposure.

## Significance Statement

This study uncovers novel aspects of nicotinic acetylcholine receptor neuropharmacology and ventral tegmental area (VTA) neurobiology that have implications for understanding nicotine dependence mechanisms. Nicotine must interact with receptors and circuits in VTA to cause dependence, and this study advances our understanding of specific nicotine-sensitive circuits that reside within this brain area. Identifying these novel nicotine-sensitive systems could provide new/additional mechanisms for targeting with smoking cessation drugs or therapeutic agents. Our results also add new details to the conceptual framework associated with reward circuit wiring, which could lead to an improved mechanistic understanding of natural reward processing.

## Introduction

Tobacco addiction is the leading cause of preventable death (Centers for Disease Control and Prevention, 2018). Although smoking rates in the United States declined from 2005 to 2017, electronic cigarette usage has increased, which may promote relapse to traditional cigarette smoking (Young-Wolff et al., 2018). Smoking cessation medications are not sufficiently effective at fostering cessation, and a better understanding of the mechanisms of nicotine dependence could lead to novel and/or more efficacious pharmacological treatment approaches.

The ventral tegmental area (VTA) is a key structure in dopamine (DA) reward circuitry, and the interaction of nicotine with VTA components is a necessary, though not sufficient, condition for nicotine dependence. Rodent models have provided substantial insight into this interaction. For example, nicotine intravenous self-administration in the rat requires functional activity of nicotinic acetylcholine receptors (nAChRs) in VTA neurons (Corrigall et al., 1994). Mice lacking the β2 nAChR are insensitive to the dopamine-stimulating and reinforcing action of nicotine (Picciotto et al., 1995, 1998), and re-expression of β2 subunits into the VTA of these mice restores nicotine modulation of VTA neuron activity and nicotine self-administration behavior (Maskos et al., 2005). Mansvelder and McGehee (2000) and Mansvelder et al. (2002) first indicated the potential for intra-VTA circuitry in the action of nicotine by showing that presynaptic α7- and β2-containing nAChRs mediate nicotine-stimulated glutamate and GABA release, respectively, onto VTA neurons. These data, coupled with a prior demonstration that VTA GABAergic neurons locally inhibit DA cells (Johnson and North, 1992a,b), helped to establish a framework to explain the direct and indirect modulation by nicotine of VTA DA neuron output.

DA, long known to be involved in natural reward and motivation to seek drugs of abuse (Lüscher and Malenka, 2011), is not the only neurotransmitter released from VTA neurons. The aforementioned VTA GABAergic neurons (1) locally inhibit VTA DA neurons (Johnson and North, 1992b; Tan et al., 2012), and (2) send long-range inhibitory projections to target structures such as nucleus accumbens (NAc; van Zessen et al., 2012) and lateral habenula (Root et al., 2014b). nAChRs, which are functionally expressed on VTA GABA neurons (Yan et al., 2018), play an important role in shaping the rewarding response to nicotine (Tolu et al., 2013; Ngolab et al., 2015). VTA DA neurons also receive asymmetric (i.e., excitatory) synaptic inputs from local glutamatergic neurons in the VTA (Dobi et al., 2010). These glutamatergic cells, which are defined by active expression of the vesicular glutamate transporter 2 (VGluT2), can directly stimulate VTA DA neurons to promote reward-like behavior (Wang et al., 2015). These various VTA cell types show preferential localization in medial VTA (mVTA) versus lateral VTA (latVTA). VTA GABAergic neurons, and especially glutamatergic neurons, are more likely to be found in mVTA (Hnasko et al., 2012; Root et al., 2014a,b; Ntamati and Lüscher, 2016; Qi et al., 2016; Yoo et al., 2016), whereas classical DAergic neurons are more abundant in latVTA (Lammel et al., 2008; Yan et al., 2018).

We recently demonstrated that β2-containing nAChRs are functionally expressed on VTA VGluT2^+^ neurons (Yan et al., 2018). Gene-editing experiments, corroborated by pharmacological data, suggest that nicotine activation—not desensitization—of those receptors drives excitatory transmission in nearby/local VTA neurons (Yan et al., 2018). However, questions remain regarding cholinergic receptor modulation of local VTA glutamatergic and GABAergic microcircuits. For example, it is unknown whether nAChRs are situated on presynaptic terminals to directly modulate fast transmitter release onto local VTA cells, or whether nAChRs act in a more indirect, polysynaptic manner. In this study, we examined glutamate and GABA transmission in local VTA circuits by expressing channelrhodopsin 2 (ChR2) in mVTA VGluT2^+^ or glutamate decarboxylase 2-positive (Gad2^+^) cells followed by recording optically evoked excitatory or inhibitory postsynaptic currents in latVTA. The ability of nicotine to modulate these circuits was examined, permitting a more detailed picture of cholinergic modulation in VTA microcircuits to emerge.

## Materials and Methods

### Materials and viral vectors

Dihydro-β-erythroidine hydrobromide (DHβE), methyllycaconitine (MLA), picrotoxin (PTX), atropine sulfate (atropine), and 4-aminopyridine (4-AP) were obtained from Sigma-Aldrich. 6-Cyano-7-nitroquinoxaline-2,3-dione (CNQX), and d(−)-2-amino-5-phosphonopentanoic acid (d-AP5) were obtained from Tocris Bioscience. QX314 chloride (QX314) was from EMD-Millipore. Tetrodotoxin (TTX) was obtained from Abcam. Nicotine hydrogen tartrate salt was obtained from Glentham Life Sciences. AAV5.EF1a.DIO.hChR2(H134R)-eYFP.WPRE.hGH was obtained from Addgene.

### Mice

All experimental protocols involving mice were reviewed and approved by an institutional animal care and use committee. Procedures also followed the guidelines for the care and use of animals provided by the National Institutes of Health Office of Laboratory Animal Welfare. All efforts were made to minimize animal distress and suffering during experimental procedures, including during the use of anesthesia. Mice were housed at 22°C on a 12 h light/dark cycle with food and water available *ad libitum*. Mice were weaned on postnatal day 21 and housed with same-sex littermates. A tail sample was taken from each mouse for genotyping via PCR. The following mouse strains were obtained from The Jackson Laboratory: VGLUT2-IRES-Cre (stock #016963); and GAD2-IRES-Cre (stock #010802). Male and female mice were used in approximately equal numbers.

### Stereotaxic surgery

Male and female mice were used for surgery starting at 8 weeks of age. Mice were initially anesthetized with an intraperitoneal injection of a ketamine/xylazine mixture (120 mg/kg ketamine, 16 mg/kg xylazine). Mice were given additional “boost” injections of ketamine (100 mg/kg, i.p.) as needed. Alternatively, some mice were anesthetized with isoflurane: 3% (flow rate, 500 ml/min) for induction, and 1.5% (flow rate, 28 ml/min) for maintenance. Mice were secured into a stereotaxic frame, and a small incision at the top of the head was made to expose the skull. Coordinates (unilateral) used for mVTA injections were as follows (relative to bregma, in mm): mediolateral, +0.01 (or −0.01); anteroposterior (AP), −3.2; dorsoventral (DV), −4.55. Exact coordinates were adjusted to account for slight differences in the head size of individual mice: the bregma/lambda distance measured for each mouse was divided by the reported bregma/lambda distance for C57 background mice (4.21), then multiplied by the AP coordinate. The injection needle was slowly lowered through the drilled hole to the DV coordinate. For adeno-associated viruses (AAVs), 500 nl of virus was infused at a rate of 50 nl/min. For all stereotaxic injections, the injection needle was left in place for 10 min after the infusion ended before slowly retracting the needle. Sutures were used to close the incision. At the conclusion of the surgery, mice were given ketoprofen (5 mg/kg, s.c.), placed in a recovery cage, kept warm, and observed until they were ambulatory. Mice were single housed following virus injection surgery and were given at least 14 d to recover and for the virus to express before beginning experimental procedures. For electrophysiology experiments, accurate targeting of the VTA was determined via direct visualization of fluorescent neurons in brain slices during recordings.

### Immunohistochemistry and confocal microscopy

Mice were anesthetized with sodium pentobarbital (200 mg/kg, i.p.) and transcardially perfused with 30 ml of PBS followed by 30 ml of 4% paraformaldehyde. Brains were dissected and postfixed in 4% paraformaldehyde overnight at 4°C. Coronal brain slices (50 μm) were cut on a freezing sliding microtome (SM2010R; Leica). VTA-containing slices were stained using the following procedure. Slices were first permeabilized for 2 min via incubation in PBST (0.3% Triton X-100 in PBS), followed by a 60 min incubation in blocking solution [0.1% Triton X-100, 5% horse serum in Tris-buffered saline (TBS)]. The primary antibodies used in this study were as follows: sheep anti-TH (catalog #AB1542; Millipore), rabbit anti-GFP (catalog #A11122; Thermo Fisher Scientific). Primary antibodies were diluted in blocking solution (anti-TH at 1:1000, anti-GFP at 1:500). Slices were incubated in primary antibodies overnight at 4°C. Three 10 min washes in TBST (0.1% Triton X-100 in TBS) were performed before transferring slices to secondary antibodies for a 60 min incubation at room temperature (donkey anti-sheep Alexa Fluor 647; chicken anti-rabbit Alexa Fluor 488, diluted to 1:500 in blocking solution). Slices were washed as before, mounted on slides, and coverslipped with VECTASHIELD.

### Brain slice preparation and recording solutions

Mice were anesthetized with Euthasol (sodium pentobarbital, 100 mg/kg; sodium phenytoin, 12.82 mg/kg) before transcardiac perfusion with an oxygenated (95% O_2_/5% CO_2_), 4°C *N*-methyl-d-glucamine (NMDG)-based recovery solution that contains the following (in mm): 93 NMDG, 2.5 KCl, 1.2 NaH_2_PO_4_, 30 NaHCO_3_, 20 HEPES, 25 glucose, 5 sodium ascorbate, 2 thiourea, 3 sodium pyruvate, 10 MgSO_4_ · 7H_2_O, and 0.5 CaCl_2_ · 2H_2_O, at 300–310 mOsm and pH 7.3–7.4. Brains were immediately dissected after the perfusion and held in an oxygenated 4°C recovery solution for 1 min before cutting a brain block containing the VTA and sectioning the brain with a vibratome (VT1200S; Leica). Coronal slices (200–250 μm) were sectioned through the VTA and transferred to an oxygenated 33°C recovery solution for 12 min. Slices were then kept in holding solution containing the following (in mm): 92 NaCl, 2.5 KCl, 1.2 NaH_2_PO_4_, 30 NaHCO_3_, 20 HEPES, 25 glucose, 5 sodium ascorbate, 2 thiourea, 3 sodium pyruvate, 2 MgSO_4_ · 7H_2_O, and 2 CaCl_2_ · 2H_2_O, at 300–310 mOsm and pH 7.3–7.4 for ≥60 min before recordings. Brain slices were transferred to a recording chamber (1 ml volume) being continuously superfused at a rate of 1.5–2.0 ml/min with oxygenated 32°C recording solution. The recording solution contained the following (in mm): 124 NaCl, 2.5 KCl, 1.2 NaH_2_PO_4_, 24 NaHCO_3_, 12.5 glucose, 2 MgSO_4_ · 7H_2_O, and 2 CaCl_2_ · 2H_2_O, at 300–310 mOsm and pH 7.3–7.4. For all recordings, the recording solution was supplemented with 1 μm atropine to eliminate contributions from muscarinic ACh receptors. Patch pipettes were pulled from borosilicate glass capillary tubes (1B150F-4; World Precision Instruments) using a programmable microelectrode puller (P-97; Sutter Instrument). Tip resistance ranged from 7.0 to 10.0 MΩ when filled with internal solution. A potassium gluconate-based internal solution was used for the following optical EPSC recordings (in mm): 135 potassium gluconate, 5 EGTA, 0.5 CaCl_2_, 2 MgCl_2_, 10 HEPES, 2 MgATP, and 0.1 GTP, with pH adjusted to 7.25 with Tris base and osmolarity adjusted to 290 mOsm with sucrose. A cesium-based internal solution was used for the following optical IPSC recordings (in mm): 120 CsCH_3_SO_3_, 20 HEPES, 0.4 EGTA, 2.8 NaCl, 5 TEA, 2.5 Mg-ATP, and 0.25 Na-GTP, at pH 7.25 and 290 mOsm with sucrose. Both internal solutions also contained QX314 (2 mm) for improved voltage control.

### Standard patch-clamp electrophysiology

Neurons within brain slices were first visualized with infrared or visible differential interference contrast optics, followed in some cases by fluorescence microscopy to identify neurons expressing fluorescent proteins. Electrophysiology experiments were conducted using a Nikon Eclipse FN-1 or Scientifica SliceScope. A computer running pCLAMP 10 software was used to acquire whole-cell recordings along with a Multiclamp 700B or Axopatch 200B amplifier and an analog-to-digital A/D converter (Digidata 1440A or Digidata 1550A). pCLAMP software, Multiclamp/Axopatch amplifiers, and Digidata A/D converters were from Molecular Devices. Data were sampled at 10 kHz and low-pass filtered at 1 kHz. Immediately before gigaseal formation, the junction potential between the patch pipette and the superfusion medium was nulled. Series resistance was uncompensated. A light-emitting diode (LED) light source (XCite 110LED; Excelitas) coupled to an excitation filter (470/40 nm bandpass) was used to stimulate the preparation with light flashes. Light flashes were triggered by pCLAMP via transistor–transistor logic pulses. Flash energy output from the LED was determined by calibration using a photodiode power sensor (model S120C; Thorlabs). Optical pulse duration (0.5–5 ms) and flash strength (0.01–0.1 mW/mm^2^) were empirically chosen for each cell such that baseline responses were initially ∼50–150 pA. Drugs were applied via bath superfusion. For our recording chamber (volume, 1 ml) and solution flow rate, we estimate that complete solution exchange occurs in 5–8 min.

### mRNA *in situ* hybridization and expression analysis

Mice were deeply anesthetized with Euthasol and decapitated. Brains were quickly removed on ice, snap frozen, and embedded in cryoembedding medium (OCT). Brains were sectioned on a cryostat (CM3050; Leica) into 20 μm sections, sections were adhered to Superfrost Plus slides, and kept at −20°C to dry for 60 min and stored at −80°C until use. Sections were fixed with 4% paraformaldehyde and processed for RNAscope [Advanced Cell Diagnostics (ACD; http://acdbio.com)] multichannel fluorescence *in situ* hybridization (FISH) according to the manufacturer manual for Multiplex assays. Sections were mounted with ProLong Gold Antifade Mountant with DAPI (Thermo Fisher Scientific). Probes for the detection of specific targets (*Chrnb2*, *Gad2*, *VGluT2* [*Slc17a6*]) were purchased from ACD.

Sections were imaged on a Nikon A1 confocal microscope according to the following parameters: 1024 × 1024 pixels, ∼200 nm/pixel, and 20× 0.75 numerical aperture objective. Nikon system images were processed with custom scripts in ImageJ (NIH). All images to be used for FISH quantification were acquired and processed in the same manner. FISH quantification used the “fluorescence coverage (%)” method, which reports the fraction of fluorescent pixels to total pixels in a cellular region of interest (ROI). An ImageJ script used DAPI staining to locate nuclei for automated and unbiased creation of cellular ROIs. The DAPI image was filtered with a Gaussian blur filter (σ = 3), thresholded (ImageJ “default” threshold), and the thresholded nuclei were dilated slightly (MorphoLibJ dilation filter; disk, radius = 2) to allow the capture of the RNA fluorescence signal just outside the nucleus but still presumably within the cell. A watershed algorithm was then applied to the filtered, binary DAPI image to isolate/separate adjacent nuclei. Finally, ROIs were detected in ImageJ (Analyze Particles algorithm; size = 20 to infinity; circularity = 0.5–1.0) and saved for application to fluorescence channel images. FISH channel images were each processed as follows: Gaussian blur filter (σ = 1); Mexican Hat filter (radius = 2); and threshold (Otsu algorithm). ROIs from the DAPI image routine were then applied to the filtered/thresholded FISH channel and a raw “percentage coverage” value was derived for each ROI. For each channel, these raw percentage coverage values for each ROI were then scaled to the single ROI in the dataset with the highest percentage coverage. This normalization step accounted for differences in probe performance and target gene expression levels. Using these transformed/normalized datasets, a cutoff value of 10% “normalized percentage coverage” was uniformly assigned to each distribution. A scatter plot shows this cutoff for each channel as a horizontal and vertical line at 10% normalized coverage. Exactly *n* = 3 mice were sampled, and two images of the mVTA were analyzed per mouse.

### Statistics and data analysis

The α level was set to 0.05 for all statistical tests, which were conducted with GraphPad Prism 7 (GraphPad Software). Null hypothesis statistical testing was used, where the null hypothesis stated, in general, that drug treatments have no effect on the physiologic measures being taken. We pooled all baseline (before nicotine treatment) responses to examine the underlying distribution of optical EPSC (oEPSC) and optical IPSC (oIPSC) amplitudes. Both were normally distributed, so parametric tests were selected for analysis. Statistical testing consisted either of one-tailed paired *t* test (for paired samples with an expected effect direction) or repeated-measures ANOVA for three or more paired samples. For the latter, omnibus testing was conducted to determine whether an effect of treatment existed. When omnibus test results resulted in an *F* statistic with an associated *p* value <0.05, subsequent comparisons were made to identify which treatment group differences accounted for the overall main effect uncovered by the omnibus test. For such *post hoc* comparisons, specific comparisons were made instead of comparing all groups with all other groups. This choice necessitated using the Sidak multiple-comparisons test. Effect sizes and *post hoc* power determinations were conducted with G*Power 3.1 or R. Bootstrap 95% confidence intervals (CIs) for the mean difference were determine in R using the “dabestr” package. Image analysis was performed with ImageJ (NIH). Analysis of electrophysiology data were performed with Clampfit (Molecular Devices) and custom scripts written in MATLAB (MathWorks). Throughout the figure legends, the number of individual neurons tested is stated immediately before the number of animals from which those neurons were derived. Nicotine-mediated increases or decreases in oEPSC/oIPSC amplitude were assessed as follows. Several responses (typically, approximately four) were recorded and averaged before and after nicotine bath application. When this mean response after nicotine exceeded the pre-nicotine application mean, the cell was classified as having an increased response. Conversely, cells with a post-nicotine application mean that was less than the pre-nicotine application mean were classified as having a decreased response. No cells demonstrated an equal response before and after nicotine application.

## Results

**Figure 1.**
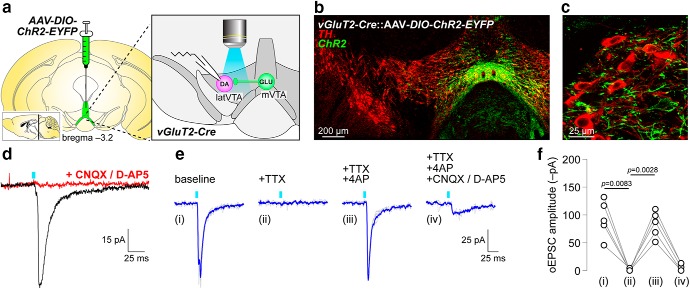
mVTA to latVTA glutamate transmission is monosynaptic. ***a***, AAV-DIO-ChR2-EYFP vectors were unilaterally microinjected into mVTA of VGluT2-Cre mice to permit optical stimulation of mVTA terminals in latVTA. ***b***, TH/EYFP stain shows ChR2 expression in mVTA somata. ***c***, TH/EYFP stain in latVTA shows ChR2 expression from mVTA in terminals surrounding TH^+^ cells. ***d***, oEPSCs recorded in latVTA are sensitive to CNQX (10 μm)/d-AP5 (30 μm) application. ***e***, An oEPSC trace family from a representative latVTA neuron is shown (gray, individual stimulation trials; blue, averaged trace). oEPSCs were measured at baseline (i) and following superfusion of the indicated drugs (ii–iv). ***f***, Summary before/after scatter plot for all cells studied as described in ***e***. *n* = 5 neurons from 4 mice (1 male, 3 female).

To study local glutamatergic circuits within the VTA, ChR2-enhanced yellow fluorescent protein (EYFP) was expressed in a Cre recombinase-dependent manner in medial VTA VGluT2^+^ neurons of VGluT2-Cre mice via microinjection of AAV vectors (Fig. 1*a*, left). Patch-clamp recordings were made in neurons of the latVTA, an area enriched in DA neurons (Lammel et al., 2008). ChR2-expressing terminals in latVTA from mVTA-derived glutamate neurons were activated with full-field flashes (∼470 nm; 0.01–0.1 mW/mm^2^) through the 40× microscope objective (Fig. 1*a*, exploded right panel). We verified viral targeting of EYFP-tagged ChR2 in mVTA via anti-GFP immunohistochemistry; ChR2-EYFP was strongly expressed in mVTA (Fig. 1*b*). ChR2 was also targeted to axons/fibers that were intermingled with TH^+^ DA neurons in latVTA (Fig. 1*c*). Using patch-clamp electrophysiology, we validated that ChR2 activation in mVTA-derived VGluT2^+^ terminals led to glutamate release onto latVTA neurons. Robust oEPSCs were evoked with ∼470 nm light flashes, and oEPSCs were fully sensitive to bath application of ionotropic glutamate receptor antagonists (CNQX, 10 μm; d-AP5, 30 μm; Fig. 1*d*). The connection rate was high between mVTA VGluT2^+^ and latVTA neurons: *n* = 44 latVTA neurons; *n* = 38 exhibited oEPSCs. Synaptic responses typically exhibit 2–10 ms synaptic delay (Yan et al., 2018), whereas direct ChR2-mediated photocurrents do not. Recorded oEPSCs had a synaptic delay of 6.1 ms [SD = 2.5; *n* = 20 neurons, *n* = 12 mice (7 male, 5 female)], confirming the synaptic nature of these responses. These results are the first to describe a high rate of excitatory connectivity between mVTA VGluT2^+^ and latVTA neurons.

Next, we asked whether mVTA-to-latVTA excitatory transmission is monosynaptic or polysynaptic. These can be distinguished by first blocking activity-dependent oEPSCs with TTX (0.5 μm), followed by coapplication of TTX with K^+^ channel blocker 4-AP (100 μm). If connections are monosynaptic, 4-AP will typically surmount the TTX-mediated block of oEPSCs. Last, CNQX/d-AP5 was applied to determine whether the responses were mediated by ionotropic glutamate receptors (Fig. 1*e*). In *n* = 5 latVTA neurons, this treatment sequence resulted in a main effect of treatment (*F*_(1.551,6.205)_ = 23.05; *p* = 0.0017; Table 1, “a” row), as indicated by a one-way repeated-measures ANOVA omnibus test on groups/conditions i–iii shown in Figure 1*e*. The CNQX/d-AP5 condition was not included in the statistical testing because this treatment was used as an exclusion metric; although there were no such cells, if a cell had been insensitive to CNQX/d-AP5, it would have been excluded from the analysis. Follow-up Sidak multiple-comparison testing indicated that TTX suppressed oEPSCs (*p* = 0.0083; Table 1, “b” row; Fig. 1*f*), while TTX + 4-AP promoted the recovery of the oEPSC (*p* = 0.0028; Table 1, “c” row; Fig. 1*f*). These results demonstrate that mVTA-to-latVTA glutamate transmission is very likely to be monosynaptic.

**Table 1:** Statistical table

	Fig.	Data structure	Type of test	*p* Value	Effect size**	95% CI (pA)
a	1*f*	Normal	One-way RM ANOVA	0.0017	0.8521	n/a
b	1*f*	Normal	Sidak *post hoc*	0.0083*	−92.3 pA	−37.8 to −146.8
c	1*f*	Normal	Sidak *post hoc*	0.0028*	82.5 pA	119.o to 46.0
d	2*a*	Normal	One-way RM ANOVA	0.5454	0.0395	n/a
e	2*d*	Normal	Paired *t* test (one tailed)	0.0019	14.5 pA	7.8 to 21.2
f	2*f*	Normal	Paired *t* test (one tailed)	0.0032	−20.47 pA	−32.2 to −8.7
g	3*b*	Normal	Paired *t* test (one tailed)	0.0065	14.55 pA	4.2 to 24.9
h	3*d*	Normal	Paired *t* test (one tailed)	0.2721	−1.45 pA	−7.2 to 4.3
i	5*f*	Normal	One-way RM ANOVA	0.0010	0.5563	n/a
j	5*f*	Normal	Sidak *post hoc*	0.0000026*	−60.45 pA	−43.1 to −77.9
k	5*f*	Normal	Sidak *post hoc*	0.0051*	64.45 pA	107.8 to 21.1
l	6*b*	Normal	One-way RM ANOVA	0.00000031	0.9384	n/a
m	6*b*	Normal	Sidak *post hoc*	0.000070*	−20.67 pA	−15.1 to −26.3
n	6*b*	Normal	Sidak *post hoc*	0.000069*	16.79 pA	21.3 to 12.3

Effect sizes and power were calculated with G*power 3.1. n/a, Not applicable; RM, repeated measures.

*Multiplicity-adjusted *p* value; **For RM ANOVA, effect size reported is *R* ^2^ = SS_treatment_/(SS_treatment_ + SS_residual_); for Sidak test and paired *t* test, effect size reported is the mean difference in pA.

**Figure 2.**
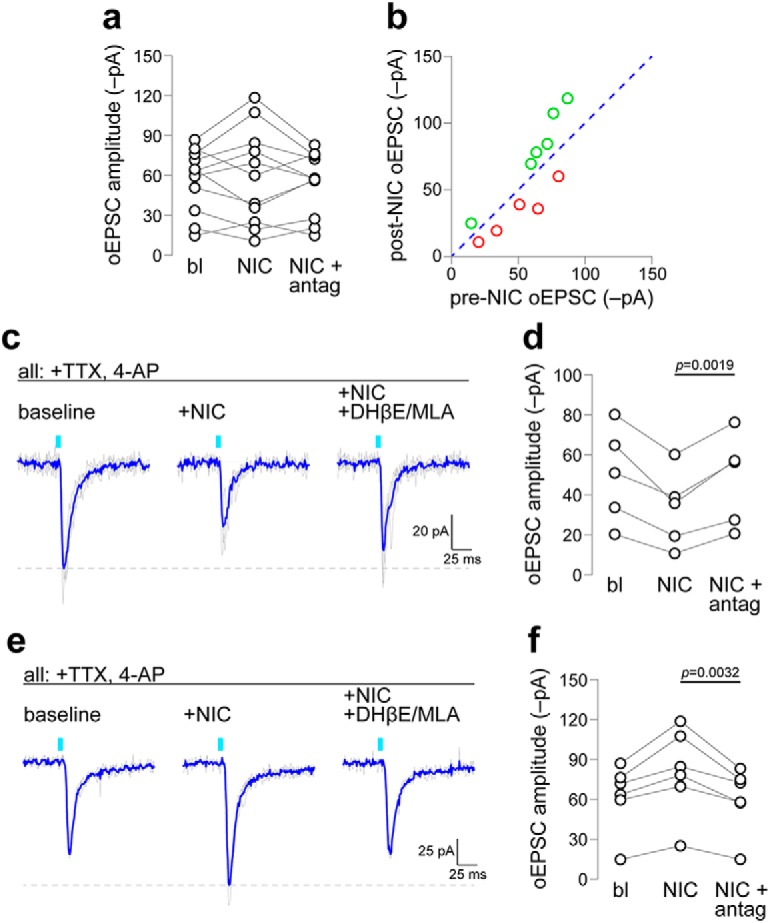
Nicotine modulation of monosynaptic mVTA-to-latVTA glutamate transmission. VGluT2^+^, ChR2-bearing fibers from mVTA were stimulated during recordings from latVTA cells at baseline, after superfusion with nicotine (0.3 μm), and following superfusion with nicotine + DhβE + MLA. ***a***, Before/after plot summarizing all tested cells. ***b***, Baseline versus nicotine data from ***a*** are plotted on an *x*–*y* plot where the blue dashed line indicates the unity line of no change. Open red circles indicate cells exhibiting a nicotine-mediated reduction, whereas open green circles indicate cells exhibiting a nicotine-mediated increase. ***c***, An oEPSC trace family is shown from a representative latVTA neuron exhibiting a nicotine-mediated decrease in oEPSC amplitude (gray, individual stimulation trials; blue, averaged trace). ***d***, Summary before/after scatter plot for all red data points indicated in ***b***. *n* = 5 neurons from 4 mice (2 male, 2 female). ***e***, An oEPSC trace family is shown from a representative latVTA neuron exhibiting a nicotine-mediated increase in oEPSC amplitude (gray, individual stimulation trials; blue, averaged trace). ***f***, Summary before/after scatter plot for all green data points indicated in ***b***. *n* = 6 neurons from 5 mice (2 male, 3 female).

Having isolated the activity of glutamatergic terminals from mVTA VGluT2^+^ cells, we next asked whether nAChR activation could modulate these monosynaptic excitatory synapses. To do this, oEPSCs recorded in latVTA neurons were first blocked by TTX and recovered with TTX + 4-AP. Nicotine (along with the continued presence of TTX + 4-AP) was then bath applied, followed by nicotine plus a nAChR antagonist cocktail (10 μm DHβE, 10 nm MLA). One-way omnibus ANOVA indicated no main effect of drug treatment (*F*_(1.054,10.54)_ = 0.4116; *p* = 0.5454; Table 1, “d” row; Fig. 2*a*). However, when the before/after data were visualized on an *x–y* plot with an associated unity line (Fig. 2*b*), it was clear that the effect of nAChR activation was bidirectional. In *n* = 5 of 11 latVTA cells [*n* = 4 mice (2 male, 2 female)], nicotine suppressed monosynaptic oEPSC amplitude (Fig. 2*c*, left and middle traces). In the other *n* = 6 of 11 cells, nicotine-enhanced monosynaptic oEPSC amplitude (Fig. 2*e*, left and middle traces). To formally examine this bidirectional effect with null hypothesis statistical testing, it was not appropriate to assign cells to these specific subgroups based on the valence of their response to nicotine and to then follow this by statistical testing taking the null hypothesis of “no effect of nicotine.” However, taking these subgroups as the reference point, we felt it appropriate to formally examine the effect of nAChR antagonist treatment compared with the “baseline” response in the presence of nicotine. In both the “nicotine decrease” and “nicotine increase” subgroups, nAChR antagonist cocktail treatment effectively increased (*t* = 6.001, df = 4, *p* = 0.0019, one-tailed paired *t* test; Table 1, “e” row; Fig. 2*d*) or decreased (*t* = 4.479, df = 5, *p* = 0.0032, one-tailed paired *t* test; Table 1, “f” row; Fig. 2*f*) the oEPSC amplitude, respectively. It should be noted that this statistical analysis approach was not planned before the experiment, as the bidirectional effect of nicotine we uncovered was not anticipated. Overall, these data indicate that nicotine has complex effects on mVTA-to-latVTA glutamate transmission; depending on the recorded cell, nicotine either enhanced or suppressed local glutamate transmission.

**Figure 3.**
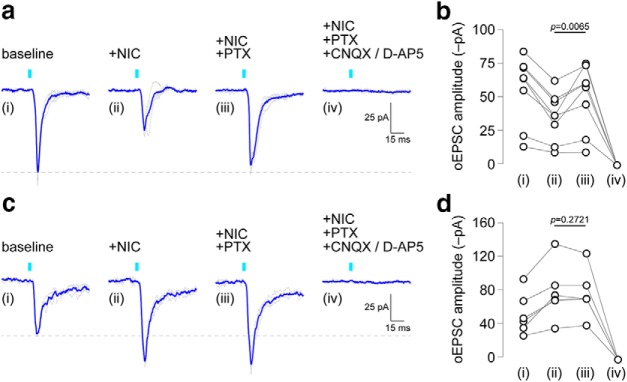
Nicotine-mediated suppression of oEPSCs occurs through GABA release. ***a***, An oEPSC trace family from a representative latVTA neuron, which exhibited nicotine-mediated suppression of the oEPSC, is shown (gray, individual stimulation trials; blue, averaged trace). oEPSCs were measured at baseline (i), after superfusion of nicotine (0.3 μm; ii), after nicotine plus PTX (50 μm; iii), and after nicotine/PTX plus CNQX (10 μm) and d-AP5 (30 μm; iv). ***b***, Summary before/after scatter plot for all cells studied as described in ***a***. *n* = 6 neurons from 4 mice (2 male, 2 female). ***c***, An oEPSC trace family from a representative latVTA neuron, which exhibited nicotine-mediated enhancement of the oEPSC, is shown (gray, individual stimulation trials; blue, averaged trace). oEPSCs were measured at baseline (i), after superfusion of nicotine (0.3 μm; ii), after nicotine plus PTX (50 μm; iii), and after nicotine/PTX plus CNQX (10 μm) and d-AP5 (30 μm; iv). ***d***, Summary before/after scatter plot for all cells studied as described in ***c***. *n* = 8 neurons from 6 mice (4 male, 2 female).

Nicotine-mediated enhancement of oEPSCs in VTA microcircuits is consistent with prior work (Yan et al., 2018), but nicotine-mediated suppression of oEPSCs (Fig. 2*c*,*d*) has not been previously reported. Based on recent published work that established the corelease of glutamate and GABA from mVTA cells (Root et al., 2014b), we speculated that the nicotine-mediated suppression of oEPSCs could indicate the corelease of GABA from synaptic terminals derived from mVTA VGluT2^+^ neurons. To test this idea, a recording was made in latVTA and an oEPSC was evoked with a ∼470 nm light flash. Nicotine was then bath applied, allowing us to identify neurons based on subgroups presented in Figure 2. To determine whether nicotine application activated GABA release to mediate the suppression or enhancement in the oEPSC, PTX was coapplied with nicotine and the oEPSC amplitude was measured again (Fig. 3*a*,*c*). Via the same rationale as above (Fig. 2), we only formally examined the effect of PTX within each subgroup using null hypothesis statistical testing. PTX reversed the effect of nicotine in the nicotine decrease subgroup (*t* = 3.311, df = 7, *p* = 0.0065, one-tailed paired *t* test; Table 1, “g” row; Fig. 3*b*), but PTX had no effect in the nicotine increase subgroup (*t* = 0.6501, df = 5, *p* = 0.2721, one-tailed paired *t* test; Table 1, “h” row; Fig. 3*d*). Coapplication of CNQX/d-AP5 with nicotine and PTX was sufficient to abolish the oEPSC (Fig. 3*b*,*d*), confirming that the oEPSC (post-nicotine/PTX application) was indeed mediated by glutamate release. The effect of CNQX/d-AP5 was not formally examined with statistical testing because this treatment was used as an exclusion metric; though there were none, if any cells had failed to respond to CNQX/d-AP5, they would have been eliminated from the analysis. Together, these results reveal bidirectional modulation of mVTA-to-latVTA glutamate transmission by nicotine, including the potential for nicotine-mediated boosting of GABA release from VGluT2^+^ axon terminals.

Within the two groups of latVTA cells we identified, namely those with a nicotine-mediated (1) oEPSC decrease and (2) oEPSC increase, we pooled responses from experiments shown in Figures 2 and 3 to conduct a subsequent analysis on effect size and its precision. In latVTA cells with a nicotine-mediated decrease in oEPSC amplitude, nicotine decreased the oEPSC amplitude by −19.1 pA. Using bootstrap resampling, we calculated the 95% confidence interval for this effect as −23.7 to −14.6. For latVTA cells where nicotine increased the oEPSC, the effect size and 95% confidence interval are 20.7 pA (95% CI, 15.7–26.8).

**Figure 4.**
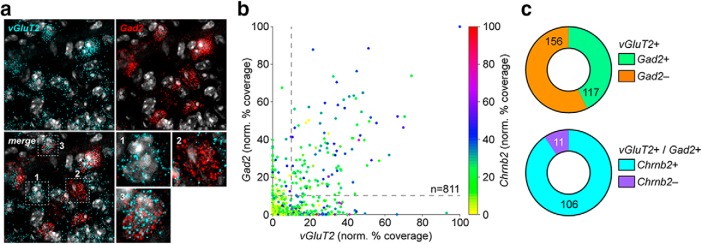
Coexpression of VGluT2 and Gad2 in mVTA. ***a***, FISH in mVTA neurons for probes: VGluT2, Gad2. Individual, representative VGluT2^+^/Gad2^−^ (1), VGluT2^−^/Gad2^+^ (2), and VGluT2^+^/Gad2^+^ (3) cells are shown (images at bottom right). ***b***, Scatter plot of VGluT2 (abscissa) versus Gad2 (ordinate) normalized percentage of coverage for all nuclei in mVTA FISH images [*n* = 3 mice (1 male, 2 female)]. Chrnb2 normalized percentage of coverage for each nucleus is represented via the indicated dot color, as defined by the scale at right. ***c***, Top, Pie graph of VGluT2^+^ nuclei showing the fraction of Gad2^+^ and Gad2^−^ nuclei. Bottom, Pie graph of VGluT2^+^/Gad2^+^ nuclei showing fraction of Chrnb2^+^ and Chrnb2^−^ nuclei.

To address the possibility of GABA release from terminals derived from VGluT2^+^ mVTA neurons, the coexpression of *VGluT2* and *Gad2* mRNA was examined in mVTA neurons using a quantitative RNA fluorescence *in situ* hybridization approach. In mVTA, we noted neurons that exclusively expressed *VGluT2*, neurons that exclusively expressed *Gad2*, and neurons that coexpressed these markers (Fig. 4*a*). Examination of *VGluT2* and *Gad2* normalized percentage coverage (a measure of transcript expression level) using a scatter plot revealed a minor but substantial subpopulation of mVTA neurons that express both markers (Fig. 4*b*). Quantification of coexpression confirmed that *n* = 117 of 273 *VGluT2* ^+^ neurons also expressed *Gad2* (Fig. 4*c*). Interestingly, nearly all mVTA *VGluT2* ^+^/*Gad2* ^+^ neurons coexpressed *Chrnb2* (β2 nAChR subunit; Fig. 4*b*,*c*), suggesting that nicotine can modulate glutamate and GABA corelease in mVTA to latVTA circuits.

**Figure 5.**
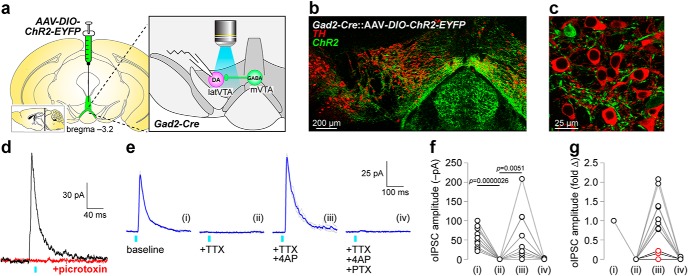
mVTA to latVTA GABA transmission is largely monosynaptic. ***a***, AAV-DIO-ChR2-EYFP vectors were unilaterally microinjected into mVTA of Gad2-Cre mice to permit optical stimulation of mVTA terminals in latVTA. ***b***, TH/EYFP stain shows ChR2 expression in mVTA somata. ***c***, TH/EYFP stain in latVTA shows ChR2 expression from mVTA in terminals surrounding TH^+^ cells. ***d***, Optical IPSCs recorded in latVTA are sensitive to PTX (50 μm) application. ***e***, An oIPSC trace family from a representative latVTA neuron is shown (gray, individual stimulation trials; blue, averaged trace). oIPSCs were measured at baseline (i) and following superfusion of the indicated drugs (ii–iv). ***f***, Summary before/after scatter plot for all cells studied as described in ***e***. *n* = 12 neurons from 7 mice (3 male, 4 female). ***g***, Data from ***f*** are shown normalized to their baseline responses. Data points in red indicate cells whose oIPSC was not recoverable with 4-AP following TTX treatment.

Next, we asked whether nicotine is also capable of modulating mVTA-to-latVTA GABA transmission. Cre-dependent AAVs directing expression of ChR2-EYFP were microinjected into the mVTA of Gad2-Cre mice, allowing optogenetic stimulation of ChR2-bearing GABAergic terminals in latVTA (Fig. 5*a*). Anti-GFP immunohistochemistry, coupled with anti-TH counterstaining, revealed strong ChR2 expression in mVTA (Fig. 5*b*). ChR2 expression was also induced in interpeduncular nucleus (IPN), consistent with previous reports of dense Gad2 expression in this structure (Zhao-Shea et al., 2013). In the latVTA of these animals, TH^+^ neurons were intermingled with—but not costained with—ChR2-EYFP^+^ GABAergic fibers (Fig. 5*c*). Optical activation of ChR2 during recordings from latVTA neurons induced robust outward currents that we refer to hereafter as oIPSCs. These oIPSCs were sensitive to PTX (Fig. 5*d*) and exhibited a synaptic delay of 6.8 ms (SD = 1.6), confirming synaptic release of GABA. The connection rate was high (*n* = 31 of 36) between mVTA GABAergic neurons and latVTA neurons.

To determine whether mVTA-to-latVTA GABA synaptic responses are monosynaptic or polysynaptic, we used the same pharmacological approach used for intra-VTA glutamate transmission. oIPSCs were blocked with TTX, then we attempted to recover them with coapplication of 4-AP with TTX before blockade of GABA_A_ receptors with PTX (Fig. 5*e*). In *n* = 13 latVTA neurons, this treatment sequence resulted in a main effect of treatment (*F*_(1.211,14.53)_ = 15.05; *p* = 0.0010; Table 1, “i” row), as indicated by a one-way repeated-measures ANOVA omnibus test. The PTX group was not included in the ANOVA because PTX sensitivity was used as an exclusion metric; PTX-insensitive responses would have been excluded from the analysis. Follow-up Sidak multiple-comparison testing indicated that TTX suppressed oIPSCs (*p* = 0.0000026; Table 1, “j” row), whereas 4-AP + TTX allowed oIPSC recovery (*p* = 0.0051; Table 1, “k” row). PTX eliminated oIPSCs in all cells tested (Fig. 5*f*). Normalizing the responses to pre-drug application levels (Fig. 5*g*) indicated that a majority of cells/responses could be fully recovered with 4-AP after TTX blockade (Fig. 5*g*, black symbols), but there were a minority of responses that did not recover (Fig. 5*g*, red symbols), suggestive of polysynaptic transmission in these cells. Nevertheless, these results are broadly suggestive that mVTA GABA neurons directly synapse onto latVTA neurons and release GABA, activating GABA_A_ receptors.

**Figure 6.**
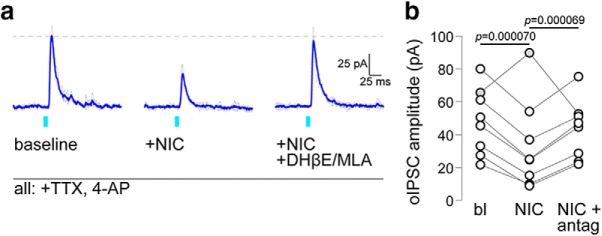
Nicotine modulation of monosynaptic mVTA to latVTA GABA transmission. ***a***, An oIPSC trace family from a representative latVTA neuron is shown (gray, individual stimulation trials; blue, averaged trace). oIPSCs were measured at baseline and following superfusion of nicotine, then nicotine + DhβE + MLA. ***b***, Summary before/after scatter plot for all cells studied, as described in ***a***. The cell indicated by gray circles was excluded as an outlier from statistical analysis. *n* = 8 neurons (1 of 8 excluded) from 6 mice (3 male, 3 female).

Finally, we studied nicotine modulation of mVTA-to-latVTA GABA transmission by isolating monosynaptic GABA responses in latVTA cells, followed by nicotine bath application, followed by bath application of nicotine plus nAChR antagonists (Fig. 6*a*). In *n* = 8 cells, 7 cells exhibited a reduction in oIPSC amplitude following nicotine application, while 1 cell exhibited an increase in oIPSC amplitude (Fig. 6*b*). In the following statistical analysis, the lone cell exhibiting a nicotine-mediated increase was excluded as an outlier after applying the ROUT method (Motulsky and Brown, 2006; maximum false discovery rate set to 1%) to a data vector containing the difference between nicotine and baseline for the *n* = 8 cell sample. The treatment sequence shown in Figure 6*a* resulted in a main effect of treatment (*F*_(1.759,10.55)_ = 91.44; *p* = 0.00000031; Table 1, “l” row), as indicated by a one-way repeated-measures ANOVA omnibus test. Nicotine induced a reduction in oIPSC amplitude (*p* = 0.00007; Table 1, “m” row), which was recoverable with coapplication of nicotine with a nAChR antagonist cocktail (10 μm DHβE, 10 nm MLA; *p* = 0.000069; Table 1, “n” row; Fig. 6*b*). These data suggest that the primary action of nicotine on mVTA-to-latVTA GABA transmission is to limit GABA release.

## Discussion

Here, we report that mVTA-to-latVTA glutamatergic and GABAergic fast synaptic transmission are largely monosynaptic and exhibit strong/reliable connectivity. Smoking-relevant concentrations of nicotine (300 nm) have differential effects on these systems; glutamate release is potentiated versus inhibited in approximately equal proportions, whereas GABA release is almost uniformly inhibited. Curiously, glutamate responses that are inhibited by nicotine show evidence of GABA corelease from VGluT2^+^ axons. Together, these results reveal a more complex system of VTA microcircuits than were previously understood. The actions of nicotine on these circuits are also complex, but collectively the data point to a net excitatory effect of nicotine on local fast synaptic transmission in the VTA.

### Physiology of local VTA circuits

We sought to record local glutamatergic and/or GABAergic synaptic transmission in VTA DA neurons. The lateral VTA, defined mainly as lateral portions of the parabrachial pigmented nucleus, is composed largely of TH^+^ DA neurons that project mainly to the lateral shell of NAc (Lammel et al., 2008, 2011). Compared with other VTA neuron subtypes, latVTA neurons most closely resemble classical substantia nigra compacta (SNc) DA neurons (Lammel et al., 2008). Based on our recent work (Yan et al., 2018), we estimate that 65.6% (*n* = 2100 TH^+^ cells of 3203 total cells analyzed) of latVTA neurons are TH^+^ DA neurons. Since this analysis was conducted on all nuclei in latVTA, which may include glial cells, this figure probably underestimates the prevalence of DA neurons relative to non-DA neurons in latVTA. Nevertheless, this indicates that a small minority of cells we recorded from in latVTA are likely to be non-DAergic. Our results indicate a high rate of connectivity between mVTA-derived fibers (glutamatergic or GABAergic) and latVTA somata/dendrites. For glutamatergic connections, this is consistent with a prior study that reported 100% connectivity between the recorded neuron and local VTA glutamatergic fibers (Yoo et al., 2016). Note, however, that this study did not specify the location within the VTA of the recorded neuron (e.g., mVTA, latVTA).

These results extend data reported previously and provoke new questions. Recently, it was demonstrated that NAc input to latVTA and mVTA neurons is primarily from the lateral versus the medial NAc shell, respectively (Yang et al., 2018). Reciprocal connections from VTA to NAc are similarly constrained into lateral or medial streams of information flow. In light of this apparent insulation of mVTA to medial NAc shell circuits from latVTA to lateral NAc shell circuits, how does the cross talk between mVTA and latVTA that we report modulate information flow between the VTA and forebrain targets like NAc or lateral habenula (Root et al., 2014a)? How does endogenous acetylcholine further modulate this cross talk? Subsequent studies will be required to address these issues.

### Nicotine modulation of local VTA circuits

In this study, we show that nicotine concentrations relevant to tobacco consumption (300 nm; Matta et al., 2007) can bidirectionally modulate local glutamate release in mVTA-to-latVTA synaptic circuits. The overall effect sizes measured from pooled subgroup (nicotine-mediated excitation and suppression) data, along with their associated 95% confidence intervals calculated via bootstrap resampling, reveal a modest yet reliably consistent dual action of nicotine. Among synapses responding to nicotine with a decrease in oEPSC amplitude, the mean decrease was 19.1 pA (decrease 95% CI, 14.6–23.7]. Considering the baseline amplitude, this amounts to a biologically meaningful reduction in glutamate release of ∼40%. Likewise, responses to nicotine with the stimulation of glutamate release (20.7 pA; 95% CI, 15.7–26.8) also point to a biologically meaningful ∼33% increase in glutamate release. Combined with a more uniform inhibition of mVTA-to-latVTA GABA release by nicotine, the net action of nicotine on local circuits impinging on latVTA could be excitatory; GABA release is dampened while glutamate release is reduced in ∼50% of latVTA cells and is enhanced in the other ∼50%. This collective stimulation of latVTA by nicotine will presumably enhance burst firing in DA neurons (Mameli-Engvall et al., 2006) and stimulate DA release in NAc, contributing to the psychostimulant and rewarding properties of nicotine. This conclusion would be dependent, however, on the relative amount of local excitatory versus inhibitory innervation of latVTA neurons.

Using pharmacology coupled with whole-cell recordings, we demonstrate the definitive existence of presynaptic nAChRs in local mVTA-to-latVTA glutamate and GABA circuits. Although our usage of a β2 and α7 nAChR antagonist cocktail in the present study precludes the determination of the nAChR subtype that modulates transmitter release, prior CRISPR (clustered regularly interspaced short palindromic repeats)-mediated knockdown of β2 subunit expression suggests that heteromeric nAChRs play a key role (Yan et al., 2018). Does nicotine modulation of local VTA glutamate and GABA imply that endogenous ACh also modulates the release of these transmitters? Based on dramatically different pharmacokinetics/pharmacodynamics between nicotine in tobacco products and endogenous ACh (Wathey et al., 1979; Papke, 2014), the latter may not be as efficacious or potent at modulating fast transmitter release as the former.

Previous pioneering studies in VTA reported that the activation of presynaptic nAChRs enhances glutamate (Mansvelder and McGehee, 2000) or GABA (Mansvelder et al., 2002) release. By contrast, we found in VTA microcircuits that nicotine suppresses glutamate or GABA release in many neurons. These seemingly unexpected findings, compared with those of Mansvelder and McGehee (2000) and Mansvelder et al. (2002), likely relate to numerous methodological and anatomic differences. In particular, the following important parameters differed between our work and the work of Mansvelder and McGehee (2000) and Mansvelder et al. (2002): animal age and species (adult mouse vs early postnatal rat), slice orientation (coronal vs horizontal), presynaptic metric (optically evoked current vs spontaneous currents), and, most importantly, circuit specificity (genetically and pharmacologically targeted mVTA-to-latVTA transmission vs forebrain-to-VTA transmission). Cell-specific manipulations and optogenetics were not available to Mansvelder and McGehee (2000) and Mansvelder et al. (2002) at the time of their important work, and it will be of interest to revisit those studies with current tools to further describe how cholinergic systems modulate neurotransmission in VTA.

What is the mechanism of nicotine-mediated suppression of glutamate and GABA release? We provide evidence supporting the following scheme: some mVTA neurons coexpress machinery for the synthesis of glutamate and GABA (Fig. 4), and nicotine stimulates a net increase in GABA release from these neurons onto latVTA target cells (Fig. 3). Corelease of glutamate and GABA has not previously been demonstrated in VTA microcircuits, but it has been shown for several other circuits whose somata reside in the VTA (Stuber et al., 2010; Stamatakis et al., 2013; Root et al., 2014a; Yoo et al., 2016). Coexpression of glutamate and GABA synthetic machinery has also been previously shown for mVTA neurons (Root et al., 2018; Mongia et al., 2019). Alternatively, it is possible that nicotine bath application causes the release of some unidentified factor that then inhibits or promotes fast transmitter release. Such a speculative mechanism would have to be activity independent, though, since our experiments with nicotine were performed in the continued presence of TTX. Prolonged exposure of VTA slices to nicotine could induce glutamate, GABA, and/or endocannabinoid release that could then act on mGluR, GABA_B_, or CB1 G-protein-coupled receptors to attenuate optical activation of VGluT2^+^ or Gad2^+^ fibers (Merrill et al., 2015; Han et al., 2017; Varani et al., 2018). It is very unlikely, however, that nicotine suppresses release by interfering with a stimulatory action of endogenous ACh. If this were the case, nAChR antagonist application would not have reversed the action of nicotine (Figs. 2*d*, 6*b*).

### Limitations and conclusions

We isolated a specific microcircuit within the VTA, but this had limitations. Cre-dependent viral expression of ChR2 was directed to mVTA of VGluT2-Cre or Gad2-Cre mice, but the diffusion of virus to nearby brain areas is possible and would influence our results if those areas had VGluT2^+^ or Gad2^+^ neurons that projected to latVTA. ChR2 virus diffusion to nearby IPN and SNc is possible, but these areas are not known to send significant glutamatergic or GABAergic afferents to latVTA. Because we used Cre expression to induce opsins in certain cells, Cre was not available to help us unequivocally identify DA neurons in latVTA. Although DA neurons are enriched in latVTA, some latVTA neurons are not DAergic and a minor portion of our results may have come from such neurons. Although we report a high percentage of latVTA cells synaptically connected to mVTA neurons, our results may underestimate the true percentage; our *ex vivo* slicing procedure likely cannot induce a connection that was not present *in vivo*, but it may sever true connections to result in latVTA neurons that appear to be unconnected. We did not investigate the potential for latVTA-to-mVTA glutamate transmission due to a significantly lower percentage of VGluT2^+^ neurons in latVTA compared with mVTA (Taylor et al., 2014; Yan et al., 2018). Finally, conclusions about results derived from optogenetic isolation of circuits should be drawn with caution. ChR2 is an excellent tool for robust (and sometimes nonphysiologic) activation of neurons/circuits, but seemingly straightforward optogenetic results may produce a clearer picture of the underlying biology than actually exists.

In summary, this work adds to the important and emerging idea that nicotine modulates circuits wholly within the VTA in addition to afferents derived from outside the VTA. This reinforces the notion that nicotine has complex actions on VTA neurons and nAChRs, at both the somatodendritic and presynaptic location.

## Synthesis

Reviewing Editor: Lorna Role, NINDS Decisions are customarily a result of the Reviewing Editor and the peer reviewers coming together and discussing their recommendations until a consensus is reached. When revisions are invited, a fact-based synthesis statement explaining their decision and outlining what is needed to prepare a revision will be listed below. The following reviewer(s) agreed to reveal their identity: Feng Luo, John Dani.

The manuscript describes nicotine effects on fast synaptic transmission within VTA microcircuitry. The authors took advantage of Cre-transgenic mice to perform optogenetic activation of glutamatergic and GABAergic populations of VTA neurons. Measuring optogenetically evoked post-synaptic currents in lateral VTA neurons, the authors conclude that bath application of nicotine differentially modulates presynaptic inputs.

This study is valuable, and it will potentially contribute to our understanding of nicotine's effects on midbrain circuits. However, there are several concerns that will need to be addressed to properly assess the interpretations provided by the authors. As such, the reviewers had multiple suggestions for improving the final version of the manuscript, including one set of “must do” studies. The required additional work and the suggested modifications that could be confined to more detailed discussion of relevant issues are indicated as “Major issues to be addressed”. In addition to these essential issues, the reviewers had numerous suggestions for clarification and edits to the manuscript that we hope will be helpful in the revision process. These, too, are delineated below (under “important additional suggestions” and “minor issues”, respectively.

Major issues to be addressed:

1. Major Issue: Proper identification of dopamine neurons.

It is standard practice to identify DA neurons using methods beyond their appearance.

The authors must verify that they are studying DA neurons by using backfills and TH labeling or by using one of several rodent models that label TH- or DAT-positive neurons.

Specifically: As Fig.6 shows, the lateral VTA contains significant numbers of GAD2+ neurons, and it is possible that some of the recorded neurons in Figs 1-3 were GABAergic. The authors have to provide evidence that they are recording from DA neurons, such as backfills and TH labeling, along with the electrophysiological identification of dopamine neurons in the lateral VTA.

2. Major issue: Use of bath application of nicotine: effects on activation vs desensitization of specific nAChR subtypes.

The authors should make some effort to verify the extent of nAChR-subtype activation versus desensitization during their nicotinic-agonist wash-in. In general, bath application of nicotine does not offer much control, and it may be worth considering other approaches to interpret the data. Additional experimentation would be best, but some insight could be provided simply by an examination of the literature and integration of prior findings into a well-considered discussion section.

Specifically: The authors bath-applied nicotine, and that will lead to a time dependent activation vs desensitization of various nAChR subtypes. The level of activation vs desensitization will change with time until a steady-state is approached with some nAChR subtypes being mostly desensitized and others predominating depending on exactly when the measurements are made relative to the nicotine application. These issues have been examined previously and are discussed extensively in the literature. At a minimum the authors should offer a careful analysis of their findings in the context of prior reported results.

At the very least, the authors should show a time course of EPSC and IPSC changes following nicotine application and should explore the question of activation vs desensitization as different nAChR subtypes predominate at different times during the nicotine application. Related to this exploration of nicotine's effects, the authors should provide information on the bath size and exchange in the methods section concerning the duration and exchange of nicotine during the wash-in and recordings.

Important additional suggestions: (in order of appearance)

1. The introduction does not optimally set the stage for how the current study might contribute to a better understanding of nicotine effects on VTA microcircuitry. The more general statements about tobacco addiction can be made more concise and/or may be better as part of the discussion. It would be best if the introduction provided more background information on VTA circuits (e.g. lateral vs medial subdivisions) and a summary of prior work on the action of nicotine on VTA neurons/circuits to contextualize the current findings.

2. Figure 1 statistical tests require modification. A multiple comparison among TTX, TTX/4AP and TTX/4AP/CNQX/D-AP5 groups), RM-ANOVA, instead of paired-t test, should be used. This includes Figure 1e, Figure 2b&e, Figure 3b&d, Figure 6 e and Figure 7b.

3. In the example trace, Fig 1d (iv), a residual current is observed in the presence of ionotropic glutamate receptor antagonists despite the claim that the antagonists “eliminated the oEPSC in all cases...” This current -- compare residual response to the light stimulation in d(iv) vs. d(ii) -- is significant, and it would be valuable to know what it is and how it may be impinging on the interpretations.

4. The authors state that the viral expression of ChR2 diffused to brain areas around the medial VTA. For example, Figure 6 demonstrates significant ChR2 expression in the lateral VTA, SN and IPN. The first two brain areas are known to provide inhibitory input onto lateral VTA dopamine neurons and IPN GABA neurons may project to the lateral VTA as well. Therefore, it is hard to conclude that the inhibitory input recorded in the lateral VTA was mediated solely by medial VTA GABA neurons. The authors should address this potential confound. Experimentally this could be addressed by injecting smaller amounts of the virus to minimize ChR2 spread and, then, verifying that the results arise from ChR2 only in the medial VTA. Alternatively, injections directed into specific nearby areas could be shown to not produce the same results as VTA targeted injections.

5. In order to support the conclusion that nicotine induced GABA co-release from vGluT2+ fibers in latVTA, the authors wanted to show oEPSC as well as oIPSC from the same neurons. Although the authors acknowledge the difficulty of recordings from latVTA neurons that exhibit a GABA-like outward current at a holding potential of 0 mV, the statistics from the 4 cells obtained did not reveal a significant difference and as such, no conclusion can be drawn. Reaching sufficient power would be essential to address whether a subpopulation of mVTA neurons are capable of co-releasing glutamate and GABA which act on postsynaptic latVTA neurons.

Minor/ additional issues:

1. Insets in Figures 1 and 6 do not correspond exactly to the square highlighted in the low-magnification image.

2. Why did the authors use different intracellular solutions between stimulation of glutamate and GABA release?

3. Some abbreviations were not spelled out: eg, PTX.

4. P4, line 12: ability to modulate these “neural transmissions”?

5. P11, line 17: Synaptic responses typically exhibit 2-10 ms delay..... reference?

6. P11, line 18-19: “38 neurons showed oEPSCs”. Why were only 20 neurons included in the stats?

7. P12, paragraph 2. P13, line 2: What are the criteria for classifying a response as either suppression or enhancement of the oEPSC? Were there any neurons that showed no changes?

8. P15, paragraph 2: did you try to get any oEPSC from those neurons?

9. P16, line 21-24: redundant, or move to limitation.

10. P17, paragraph 3: can one be more precise about what is meant by “long whole cell recordings”?

## Author Response

Synthesis of Reviews:

The manuscript describes nicotine effects on fast synaptic transmission within VTA microcircuitry. The authors took advantage of Cre-transgenic mice to perform optogenetic activation of glutamatergic and GABAergic populations of VTA neurons. Measuring optogenetically evoked post-synaptic currents in lateral VTA neurons, the authors conclude that bath application of nicotine differentially modulates presynaptic inputs.

This study is valuable, and it will potentially contribute to our understanding of nicotine's effects on midbrain circuits. However, there are several concerns that will need to be addressed to properly assess the interpretations provided by the authors. As such, the reviewers had multiple suggestions for improving the final version of the manuscript, including one set of “must do” studies. The required additional work and the suggested modifications that could be confined to more detailed discussion of relevant issues are indicated as “Major issues to be addressed”. In addition to these essential issues, the reviewers had numerous suggestions for clarification and edits to the manuscript that we hope will be helpful in the revision process. These, too, are delineated below (under “important additional suggestions” and “minor issues”, respectively.

Major Issues

1: Proper identification of DA neurons

Comment:

It is standard practice to identify DA neurons using methods beyond their appearance.

The authors must verify that they are studying DA neurons by using backfills and TH labeling or by using one of several rodent models that label TH- or DAT-positive neurons.

Response:

We agree that it is important to discriminate DA from non-DA VTA neurons, with the caveat that our conclusions about synaptic mechanisms do not rely on the neurochemical phenotype of the target cells in latVTA. We have addressed this issue in the following way:

Our prior paper (Yan et al., Cell Reports 23, 2236-44) disclosed several large data sets utilizing quantitative RNA in situ hybridization to characterize the neurochemical phenotype of latVTA neurons. We have referenced these data in the revised manuscript, where we now give an estimate of the fraction of latVTA neurons that are expected to be DAergic (which is 65.6%). These data are resultant from an unbiased analysis of all latVTA cells/nuclei, across over 3000 cells. This approach probably provides a more accurate analysis of latVTA DA neurons than a recording/backfilling approach on a small sample size (n=10 neurons, for example), which was suggested by one or more reviewers. Apologies that this disclosure requires that we reveal our identity, but the alternative would be for us to repeat our prior work, which would have ethical implications with regard to animal usage.

Comment:

Specifically: As Fig.6 shows, the lateral VTA contains significant numbers of GAD2+ neurons, and it is possible that some of the recorded neurons in Figs 1-3 were GABAergic. The authors have to provide evidence that they are recording from DA neurons, such as backfills and TH labeling, along with the electrophysiological identification of dopamine neurons in the lateral VTA.

Response:

We respectfully disagree that Figure 6b shows a significant number of GAD2+ neurons in latVTA. ChR2+ green signal is primarily an axonal/fiber pattern rather than infected somata. Such fibers would be expected to originate from infected mVTA cell bodies. We acknowledge that we probably wrote the paper with a focus on DA neurons. In the revised manuscript, we have modified our reference to latVTA to refer generally to “latVTA neurons” rather than specifically to DA neurons.

2: Bath application of nicotine

Comment:

The authors should make some effort to verify the extent of nAChR-subtype activation versus desensitization during their nicotinic-agonist wash-in. In general, bath application of nicotine does not offer much control, and it may be worth considering other approaches to interpret the data. Additional experimentation would be best, but some insight could be provided simply by an examination of the literature and integration of prior findings into a well-considered discussion section.

Response:

Good points and thank you for raising them. Our data show that the nicotine-mediated modulation of transmitter release is reversed by co-application of a nAChR antagonist cocktail with nicotine. This is strong evidence in favor of the idea that nAChR activation, not desensitization, is responsible for the nicotine modulation. If it were desensitization mediating the effect of nicotine, we should have seen the antagonist cocktail have no effect or even to potentiate nicotine's action. Regarding prior findings, please see below.

Comment:

Specifically: The authors bath-applied nicotine, and that will lead to a time dependent activation vs desensitization of various nAChR subtypes. The level of activation vs desensitization will change with time until a steady-state is approached with some nAChR subtypes being mostly desensitized and others predominating depending on exactly when the measurements are made relative to the nicotine application. These issues have been examined previously and are discussed extensively in the literature. At a minimum the authors should offer a careful analysis of their findings in the context of prior reported results.

Response:

With regard to nicotine mediating desensitization vs. activation of nAChRs, the reviewer states “These issues have been examined previously and are discussed extensively in the literature.” This is true when it comes to generalized nAChR expression and subtypes in the VTA. However, this study is concerned with presynaptic nAChRs in mVTA to latVTA GLU or GABA microcircuits. There is little information available in the literature on nAChR subtype or activation/desensitization profile for this specific population of receptors. Our recent report (Yan et al., Cell Reports 23, 2236-44) demonstrated robust somatodendritic functional expression of nAChRs in mVTA vGluT2+ and Gad2+ neurons, however. We also demonstrated, using both CRISPR gene editing as well as pharmacological approaches, that β2-containing nAChRs mediate nicotine-mediated boosting of glutamate transmission within mVTA. This report provides strong evidence that nAChR activation, not desensitization, is involved in nicotine-mediated boosting of glutamate release. As requested by the reviewer, we have added this topic to the revised manuscript.

Comment:

At the very least, the authors should show a time course of EPSC and IPSC changes following nicotine application and should explore the question of activation vs desensitization as different nAChR subtypes predominate at different times during the nicotine application.

Response:

Our study aims and conclusions do not touch on the subject of nAChR subtypes (β2-containing vs. α7, for example), though our recent prior work strongly supports the idea that β2-containing nAChR activation plays a key role in glutamate release from mVTA cells. CRISPR deletion of β2 from mVTA vGluT2+ cells eliminated nicotine's ability to boost glutamate release. Further, pre-application of DHβE (β2 nAChR antagonist) blocked nicotine's ability to boost glutamate release. We do not have time course data available. Generating new data would require extensive experimentation. Consider that 3 different nicotine-sensitive synapses were identified in this paper: 1-boosting of GLU release from vGluT2+ cell, 2-suppression of GLU release from vGluT2+ cells, and 3-suppression of GABA release from GAD2+ cells. To examine even 2 nAChR subtypes (β2 vs α7), each of these 3 synapses would need to be probed with 2 different treatment protocols (i.e. separate groups): 1-MLA then DHβE, and 2-DHβE then MLA. These are laudable and interesting experiments to do, but we will have to save them for a subsequent paper.

Comment:

Related to this exploration of nicotine's effects, the authors should provide information on the bath size and exchange in the methods section concerning the duration and exchange of nicotine during the wash-in and recordings.

Response:

This information is provided in the revised manuscript.

Important additional suggestions

Comment:

The introduction does not optimally set the stage for how the current study might contribute to a better understanding of nicotine effects on VTA microcircuitry. The more general statements about tobacco addiction can be made more concise and/or may be better as part of the discussion. It would be best if the introduction provided more background information on VTA circuits (e.g. lateral vs medial subdivisions) and a summary of prior work on the action of nicotine on VTA neurons/circuits to contextualize the current findings.

Response:

Thank you for the suggestion. We made significant changes to the introduction along the lines suggested by the reviewer. During this re-write, we realized that the double-blind nature of eNeuro peer review (which made it impossible to self-cite other than with a very oblique reference) hindered our ability to properly contextualize our current findings because we could not adequately refer to our recent work without revealing our identity. To properly write the revised introduction as suggested by the reviewer, we have explicitly referred to our recent work to more fully prepare the reader for the coming results.

Comment:

Figure 1 statistical tests require modification. A multiple comparison among TTX, TTX/4AP and TTX/4AP/CNQX/D-AP5 groups), RM-ANOVA, instead of paired-t test, should be used. This includes Figure 1e, Figure 2b&e, Figure 3b&d, Figure 6 e and Figure 7b.

Response:

Thank you for the suggestion to improve our statistical analysis. We gave this much thought, resulting in the following changes in the revised manuscript.

Figure 1e: a one-way RM-ANOVA was used for the first 3 groups since the hypothesis tested was that these synapses are monosynaptic. CNQX/D-AP5 was used as an exclusion metric, meaning the data would have been rejected if the response failed to be blocked by this treatment. It would therefore violate the assumption of random sampling to employ an ANOVA with the CNQX/D-AP5 group included.

Figure 2: With respect to the way these data were analyzed in the original manuscript, we realized it was circular reasoning to assign cells to sub-groups based on the valence of their response to nicotine, then to conduct a statistical test that, by definition, took the null hypothesis (no effect of nicotine) as the status quo. The random sampling assumption was also violated via this method because the data in the two subgroups were not randomly selected from a representative portion of the population. In the revised manuscript, we have reported a RM-ANOVA with all cells combined (which of course had a p value > 0.05), and have demonstrated the existence of 2 types of synapses by simply reporting the grouped responses without statistical testing. In Figure 3, we have further examined and discussed the effect size and its precision for the two subgroups, in keeping with eNeuro's recent laudable announcement/suggestion that “estimation statistics” go alongside null hypothesis statistical testing. This is new analysis relative to the original manuscript.

Within these subgroups, however, we used a paired t-test to formally compare the “nicotine baseline” with the response to nicotine+antagonists, asking whether this treatment reverses the action of nicotine.

Figure 3: By the same rationale as that stated for Figure 2, we did not include the baseline vs. nicotine-mediated increase/decrease in the statistical testing in Figure 3. Similarly, CNQX/D-AP5 responses were not included either again because this was used as an exclusion metric. For Figure 3, the question being asked is whether the effect of nicotine can be reversed by PTX. Therefore, we opted for a paired t-test between the “with nicotine” baseline oEPSC and the “with nicotine plus PTX” test condition.

Figure 5e (formerly 6e): By the same rationale as in Figure 1e, we did not include the final PTX group in the revised analysis because it was used as an exclusion metric; cells insensitive to PTX would have been dropped from the analysis. RM-ANOVA with post-hoc testing was conducted in the revision, as suggested.

Figure 7b: A RM-ANOVA was employed on the 3 groups. As originally indicated, the cell in grey was excluded as an outlier. In the revised manuscript, we have been more explicit about how this outlier exclusion was conducted.

Comment:

In the example trace, Fig 1d (iv), a residual current is observed in the presence of ionotropic glutamate receptor antagonists despite the claim that the antagonists “eliminated the oEPSC in all cases...” This current -- compare residual response to the light stimulation in d(iv) vs. d(ii) -- is significant, and it would be valuable to know what it is and how it may be impinging on the interpretations.

Response:

Figure 1d show all traces from a single cell. To be transparent and to avoid exaggerating our results, we wanted to show responses from a cell that was neither the largest nor the smallest response among all tested cells. With 5 cells in the analysis, we did not consider the largest and smallest responses, leaving us with 3 cells to select from. The response family shown in panel d did indeed show a residual current. We do not know what this current is. We have modified the statement about elimination of oEPSCs to be more conservative. We point to our previous study that examined glutamate release from mVTA cells, where NBQX/D-AP5 provided full block of oEPSCs (Yan et al., Cell Reports 23, 2236-44; Fig. 4B). The incomplete block in the present manuscript may be an anomaly resultant from nothing other than random sampling. Other speculative explanations are possible too.

Comment:

The authors state that the viral expression of ChR2 diffused to brain areas around the medial VTA. For example, Figure 6 demonstrates significant ChR2 expression in the lateral VTA, SN and IPN. The first two brain areas are known to provide inhibitory input onto lateral VTA dopamine neurons and IPN GABA neurons may project to the lateral VTA as well. Therefore, it is hard to conclude that the inhibitory input recorded in the lateral VTA was mediated solely by medial VTA GABA neurons. The authors should address this potential confound. Experimentally this could be addressed by injecting smaller amounts of the virus to minimize ChR2 spread and, then, verifying that the results arise from ChR2 only in the medial VTA. Alternatively, injections directed into specific nearby areas could be shown to not produce the same results as VTA targeted injections.

Response:

As the reviewer is aware, ChR2 is primarily trafficked to axons and presynaptic terminals and its localization in somata tends to be weak relative to its localization in axons. Indeed, many investigators actually use ChR2 not for its light-activated ion channel properties but rather as a genetically-targeted anterograde tracer. So, we expect to see significant ChR2 expression in lateral VTA if mVTA sends a projection there. We are not aware of strong evidence in the literature supporting the notion that SN and/or IPN send GABAergic projections to latVTA. We have so stated in the discussion section.

Comment:

In order to support the conclusion that nicotine induced GABA co-release from vGluT2+ fibers in latVTA, the authors wanted to show oEPSC as well as oIPSC from the same neurons. Although the authors acknowledge the difficulty of recordings from latVTA neurons that exhibit a GABA-like outward current at a holding potential of 0 mV, the statistics from the 4 cells obtained did not reveal a significant difference and as such, no conclusion can be drawn. Reaching sufficient power would be essential to address whether a subpopulation of mVTA neurons are capable of co-releasing glutamate and GABA which act on postsynaptic latVTA neurons.

Response:

We have removed this experiment/result/figure from the revised manuscript due to our inability to acquire a sample size sufficient to allow for a conclusion to be drawn.

Minor/additional issues

Comment:

Insets in Figures 1 and 6 do not correspond exactly to the square highlighted in the low-magnification image.

Response:

This is true. For the sake of accuracy and transparency, we have made the insets their own panels in the revised manuscript. We have removed the dashed-line boxes to avoid confusion.

Comment:

Why did the authors use different intracellular solutions between stimulation of glutamate and GABA release?

Response:

Compared to the intracellular solution for glutamate release, a different intracellular chloride concentration is needed to more effectively measure GABA release in the postsynaptic cell.

Comment:

Some abbreviations were not spelled out: eg, PTX.

Response:

In the revised manuscript, picrotoxin has been spelled out at its first instance.

Comment:

P4, line 12: ability to modulate these “neural transmissions”?

Response:

Thank you for the suggestion, but we have retained the original wording.

Comment:

P11, line 17: Synaptic responses typically exhibit 2-10 ms delay..... reference?

Response:

A reference has been added.

Comment:

P11, line 18-19: “38 neurons showed oEPSCs”. Why were only 20 neurons included in the stats?

Response:

Some experiments involved immediate bath application of TTX/4AP before a cell was patched, whereas others involved making a baseline oEPSC recording prior to application of TTX. We could ascertain whether a cell was synaptically connected by the appearance of an oEPSC REGARDLESS of whether TTX was pre-applied, which is why we have n=38 for number of cells with an oEPSC. However, we only measured the synaptic delay in cells that did NOT have TTX pre-applied, n=20. We did not feel it was appropriate to pool synaptic delay numbers from cells that were vs. weren't exposed to TTX.

Comment:

P12, paragraph 2. P13, line 2: What are the criteria for classifying a response as either suppression or enhancement of the oEPSC? Were there any neurons that showed no changes?

Response:

We have added this classification criteria to the “Statistics and Data Analysis” section in the revised manuscript. We have also been more explicit about how the two subgroups were dealt with in the Figure 2 results section. This includes new panels in Figure 2 to more clearly show how these groupings were justified.

Comment:

P15, paragraph 2: did you try to get any oEPSC from those neurons?

Response:

No, we did not.

Comment:

P16, line 21-24: redundant, or move to limitation.

Response:

Moved as requested.

Comment:

P17, paragraph 3: can one be more precise about what is meant by “long whole cell recordings”?

Response:

Imprecise term “long” was removed.
